# Local Administrations of Iron Oxide Nanoparticles in the Prefrontal Cortex and Caudate Putamen of Rats Do Not Compromise Working Memory and Motor Activity

**DOI:** 10.1007/s12640-023-00684-x

**Published:** 2023-12-22

**Authors:** Ellen Irrsack, Sidar Aydin, Katja Bleckmann, Julia Schuller, Ralf Dringen, Michael Koch

**Affiliations:** 1 Department of Neuropharmacology, Centre for Cognitive Sciences, University of Bremen, PO Box 330440, Bremen, 28334 Germany; 2 Centre for Biomolecular Interactions Bremen (CBIB), and Centre for Environmental Research and Sustainable, Technology, University of Bremen, PO Box 330440, Bremen, 28334 Germany

**Keywords:** Iron oxide nanoparticles, Medial prefrontal cortex, Working memory, Caudate putamen, Motor activity, Immunohistochemistry

## Abstract

Iron oxide nanoparticles (IONPs) have come into focus for their use in medical applications although possible health risks for humans, especially in terms of brain functions, have not yet been fully clarified. The present study investigates the effects of IONPs on neurobehavioural functions in rats. For this purpose, we infused dimercaptosuccinic acid-coated IONPs into the medial prefrontal cortex (mPFC) and caudate putamen (CPu). Saline (VEH) and ferric ammonium citrate (FAC) were administered as controls. One- and 4-week post-surgery mPFC-infused animals were tested for their working memory performance in the delayed alternation T-maze task and in the open field (OF) for motor activity, and CPu-infused rats were tested for their motor activity in the OF. After completion of the experiments, the brains were examined histologically and immunohistochemically. We did not observe any behavioural or structural abnormalities in the rats after administration of IONPs in the mPFC and the CPu. In contrast, administration of FAC into the CPu resulted in decreased motor activity and increased the number of microglia in the mPFC. Perls’ Prussian blue staining revealed that FAC- and IONP-treated rats had more iron-containing ramified cells than VEH-treated rats, indicating iron uptake by microglia. Our results demonstrate that local infusions of IONPs into selected brain regions have no adverse impact on locomotor behaviour and working memory.

## Introduction

Iron oxide nanoparticles (IONPs) are useful for biomedical applications such as drug delivery, magnetic resonance imaging, magnetic hyperthermia and cancer theranostics (Yarjanli et al. 2017; Xie et al. 2018; Schneider et al. 2022; Vangijzegem et al. 2023). This is especially due to the magnetic properties, nanoscale structure, irreversibility of high-field magnetization, high surface area, magnetothermal effect and biocompatibility of IONPs (Xie et al. 2018; Schneider et al. 2022). However, still too little is known about potential toxic effects of IONPs on living tissue. IONPs can enter the brain across the blood–brain barrier after systemic application, via the olfactory nerve after inhalation, ingestion or directly via intracerebral administration (Wang et al. 2011a, b; Kumari et al. 2013; Gaharwar et al. 2019). Several studies report that IONPs are biocompatible and have low toxicity, while other studies show toxic effects through generation of reactive oxygen species (ROS) due to the Fenton reaction after the application of IONPs (Voinov et al. 2011; Valdiglesias et al. 2016; Mai and Hilt 2019; Vakili-Ghartavol et al. 2020). In vitro studies with cultured neurons or astrocytes showed no toxic effects of IONPs, despite of IONP-uptake (Geppert et al. 2012; Pinkernelle et al. 2012; Petters and Dringen 2014), while cultured microglia take up IONPs more efficiently leading to an impairment of cell viability (Pickard and Chari 2010; Luther et al. 2013; Petters et al. 2016). Several in vivo studies of IONPs reported histopathological and neurochemical alterations such as an increased number of activated microglia, neurodegeneration, signs of oxidative stress, damaged myelin sheaths or alterations of monoamine levels in rodent brains (Wang et al. 2007, 2009, 2011b; Wu et al. 2013; Imam et al. 2015; Askri et al. 2018; Manickam et al. 2018, 2019; Minigalieva et al. 2023). Given the potential neuropathological damage caused by IONP-administration, normal brain functioning can be compromised, which manifests, e.g. as locomotor and cognitive impairments in rodents. (Dhakshinamoorthy et al. 2017; Manickam et al. 2019; Minigalieva et al. 2023). Specifically, the striatum has been shown to be sensitive to IONP-accumulation as evidenced by increased microglia and oxidative stress (Wang et al. 2011b; Wu et al. 2013). The striatum represents an important input region of the basal ganglia and therefore plays an important role in motor control (Florio et al. 2018) and abnormalities in striatal functioning are known to cause motor deficits (Reiner and Deng 2018). Furthermore, the accumulation of IONPs in the frontal cortex led to neuronal damage and altered monoamine level, which had a detrimental effect on memory performance and motor skills in mice (Dhakshinamoorthy et al. 2017; Manickam et al. 2019). To increase stability, biocompatibility, biodegradation and solubility, IONPs receive a coating (Bardestani et al. 2021). Dimercaptosuccinic acid (DMSA) is a frequently used molecule for surface coating of IONPs which stabilises IONPs in physiological media (Fauconnier et al. 1997; Paulini et al. 2022). Furthermore, functional modifications of the coating are possible, such as binding drugs to the DMSA coat for delivery to the site of action by an external magnetic field, radiolabelling of DMSA for radionuclide therapy of solid tumours or to attach fluorescent dyes to track the fate of IONPs (Petters et al. 2016; Willmann and Dringen 2018; Martins et al. 2021; Stanković et al. 2023). Recently, we showed that intracranial applications of DMSA-coated IONPs into the medial prefrontal cortex (mPFC), caudate putamen (CPu) and dorsal hippocampus had no major adverse effects on neurons (Irrsack et al. 2021), confirming the low toxic potential of those IONPs that has been reported for cultured neurons (Petters and Dringen 2015).

As IONPs are frequently used for medical applications, it is not only important to test whether the IONPs may harm brain cells, but also to test for the functionality of the brain after IONP application by means of behavioural experiments. Therefore, the aim of this study was to investigate potential effects of injected IONPs on cognitive and motor behaviour in addition to test for altered brain histology in rats. We infused DMSA-coated IONPs into the mPFC and CPu. Saline (VEH) and ferric ammonium citrate (FAC) served as controls. Rats which received infusions into the mPFC were tested for working memory performance in a delayed-alternation T-maze task and for motor behaviour in the open field (OF), while rats which received infusions into the CPu were tested for locomotor behaviour in the OF. The animals underwent the different behavioural tests at two time points: 1-week and 4-week post-surgery. After behavioural experiments were completed, rats were sacrificed and brains were histochemically and immunohistochemically processed to assess the iron distribution, viability of neurons and activation of glial cells.

## Materials and Methods

### Iron Oxide Nanoparticles

DMSA-coated IONPs labelled with BODIPY^®^ FL C_1_ IA[N-(4,4-difluoro-5,7-dimethyl-4-bora-3a,4a-diaza-s-indacene-3-yl)methyl) iodoacetamide] were synthesised and characterized as previously described (Geppert et al. 2009; Rastedt et al. 2017). The hydrodynamic diameter of the IONPs dispersed in saline was 51.6 ± 10 nm and the ζ-potential of the IONPs was − 35.2 ± 0.4 mV (mean values ± standard deviation of two independent syntheses of IONPs). The purpose of applying IONPs labelled with BODIPY was to perform a colocalization study to test for possible uptake of IONPs in microglia, astrocytes and neurons stained via immunohistochemical methods (see below). However, no fluorescence signal could be detected from these IONPs after intracerebral infusion and immunohistological tissue processing, most likely due to adverse consequences of the used fixation and/or staining procedures on the fluorophore.

### Animals

A total of 60 naïve adult male Wistar rats (obtained from Charles River, Sulingen, Germany) were subdivided into a mPFC-group and a CPu-group (*n* = 30 each). They were housed in groups of five animals in standard Macrolon cages type IV under controlled conditions (12 h light/dark cycle, lights on at 7:00 a.m., 45–55% humidity, 21–22 °C), were food-restricted to 12 g Laboratory rodent chow (Nohrlin GmbH, Bad Salzuflen, Germany) per rat per day and had free access to drinking water.

### Intracerebral Substance Administration

Substances administered bilaterally into the mPFC or CPu were either sterile saline (0.9% NaCl) as vehicle (VEH; *n* = 10 each area), 1 mM ferric ammonium citrate (FAC; *n* = 10 each area, Roth, Karlsruhe, Germany) dissolved in sterile saline as ferric iron control (Bishop and Robinson 2001) or 1 mM iron as IONPs dispersed in sterile saline (*n* = 10 each area). The iron containing solutions were prepared freshly before use and administered in pseudorandomized order.

Rats of all groups were anaesthetized with 60 mg/kg ketamine/0.5 mg/kg medetomedine (CP-Pharma, Burgdorf, Germany) intraperitoneally (i.p.) and 0.1 mg/kg atropine (Braun, Melsungen, Germany) subcutaneously (s.c.) to support respiratory and cardiac function. Rats were fixed in a stereotaxic frame and holes were drilled bilaterally above the target positions according to the rat brain atlas of Paxinos and Watson (Paxinos and Watson 1998). The coordinates of the infusion sites for the mPFC were rostrocaudal − 2.7 mm, lateral ± 0.8 mm, ventrodorsal + 3.7 mm and for the CPu were rostrocaudal − 1.2 mm, lateral ± 2.0 mm, and ventrodorsal + 5.0 mm. Stainless 30 gauge injection cannulas connected to microliter syringes (SGE Scientific Glass Engineering, Darmstadt, Germany) via polyethylene tubes were inserted and 0.5 µL of IONPs in saline, FAC in saline or VEH were injected (0.2 µL/min). The cannula remained in the brain for additional 2.5 min to avoid substance reflux and to allow diffusion into the parenchyma. Subsequently, the cannula was withdrawn, the drill holes were closed with bone wax (SMI, Steinerberg, Belgium) and the skin was sutured.

### Behavioural Tests

**Fig. 1:**
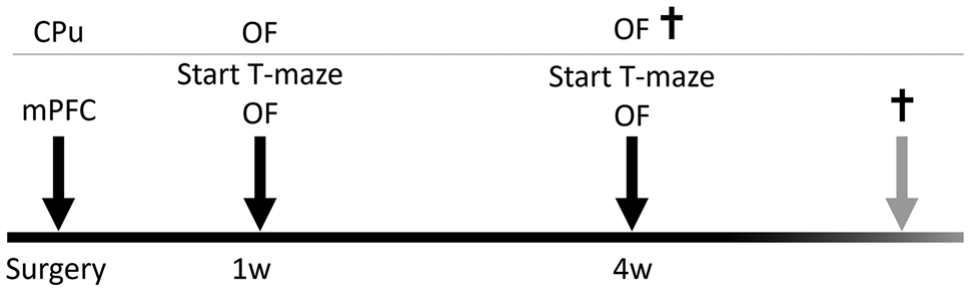
Time schedule of substance administration into the caudate putamen (CPu; *n* = 30) or medial prefrontal cortex (mPFC; *n* = 30) and behavioural testing 1-week and 4-week post-infusion. For the mPFC-group training sessions in the T-maze started 1-week and 4-week post-infusion. The open field (OF) activity was tested 1 week and 4 weeks after substance administration into the CPu and mPFC. Rats were sacrificed immediately after completion of behavioural testing, which is denoted by the cross. The time of death in the mPFC-group depended on the training performance of the animals and thus varied individually

CPu- and mPFC-groups were tested for locomotor behaviour 1-week and 4-week post-surgery in the OF. Additionally, the mPFC-group was tested for working memory performance in the delayed spatial alternation task in the T-maze starting with the training procedures 1-week and 4-week post-surgery (1-week and 4-week time-block) each concluded with a test-session (see Fig. 1).

### Spatial Delayed Alternation Task in the T-Maze

For measuring the working memory performance of the mPFC-group, rats were tested in a spatial delayed alternation task in the T-maze adapted from Brosda et al. (2011). The T-maze was constructed from black plastic and consisted of one start arm (60 × 15 × 30 cm) and two side arms (58 × 15 × 30 cm). At the end of each side arm was a 1-cm deep aluminium cup (diameter 4.5 cm) fixed to the maze floor containing the food reward (45 mg casein pellets, Bioserv, Germany). The start compartment was located at the beginning of the start arm and closed by a movable guillotine door. Prior to the training rats were habituated to the maze for 2 days. On the first day, the guillotine door was removed, and casein pellets were distributed throughout the maze. Groups of two to three rats were allowed to move freely in the maze for 10 min. On the second day, rats were placed individually into the maze. Moreover, the guillotine door was positioned at the start compartment and casein pellets were arranged in the aluminium cups. After the habituation period, rats underwent a daily training of 10 consecutive trials, where they had to alternate between the left and the right arm in order to obtain the reward. For the first trial, a reward was placed in both arms and served as free choice trial. Afterwards, rats were trained for forced-alternative choice by placing the reward in the arm opposite to the one rewarded before. Training was completed when the rats had a score of 80% correct responses for three consecutive or on four of five consecutive days. After reaching the criterion rats were tested for working memory performance by opening the guillotine doors after 0 s, 10 s and 30 s delays, and the total errors were counted.

### Open Field

Locomotor activity was tested in transparent OF boxes (44.5 × 44.5 × 44 cm, ActiMot-System, TSE-Systems, Bad Homburg, Germany). Horizontal and vertical movement and position were detected via infrared light beams. Rats were tested at dimmed illumination for 30 min. The parameters time spent in the centre [%], distance travelled [m], rearings [n] and total motor activity [%] of rats were recorded.

## Histology

### Preparation of Brain Tissue

Immediately after completion of the behavioural experiment, rats were euthanized by an overdose of pentobarbital (200 mg/kg, i.p., Sigma-Aldrich, Steinheim, Germany) and transcardially perfused with 250 mL phosphate buffered saline (PBS, 2.851% Na_2_HPO_4_ • 2H_2_O, 0.552% NaH_2_PO_4_ • H_2_O and 0.9% NaCl in aqua dest., pH 7.4) followed by 250 mL 4% paraformaldehyde (PFA; Serva Electrophoresis, Heidelberg, Germany) in 0.1 M sodium phosphate buffer (PB), pH 7.4. The brains were removed from the skull and post-fixed in 4% PFA in 0.1 M PB for 24 h, followed by cryoprotection in 30% sucrose solution (in 0.1 M PB) for 72 h. Six series of coronal brain sections (40 µm) for further histological processing were cut on a cryostat (Jung CM 3000, Leica Instrument, Nussloch, Germany).

### Nissl-Staining

To detect the infusion sites, brain sections were mounted on gelatinized slides, dried, rehydrated through a descending alcohol series, stained with thionine, dehydrated through an ascending alcohol series, and covered with Entellan^®^ (Merck, Darmstadt, Germany). The sections were examined by light microscopy and compared with standardised coronal brain sections of a rat brain atlas (Paxinos and Watson 1998).

### Staining of Tissue Sections for Iron

For detection of iron in the brain tissue, free floating brain sections were processed at room temperature (RT) by a modified staining protocol for Perls’ Prussian blue (Moos and Møllgård 1993).

Free-floating sections were incubated for 30 min in 5% potassium ferrocyanide in 0.1 M PB and then transferred for 30 min in 1:1 1% HCl and 5% potassium ferrocyanide. Thereafter, sections were washed twice for 10 min in PBS. Next, the sections were pre-incubated for 15 min in Tris-buffered saline (TBS; 1.32% tris(hydroxymethyl)aminomethane in PBS; 0.14% NaH_2_PO_4_ • H_2_O, 0.02% KCl, 0.2% NaOH and 0.8% NaCl in aqua dest., pH 7.4). For intensification of the Perls staining sections were transferred into TBS solution containing 0.05% 3,3-diaminobenzidine tetrahydrochloride and 0.07% imidazole. Afterwards, the reaction was started adding 0.3% ammonium nickel sulphate and 0.01% H_2_O_2_ for 10 min. The reaction was terminated by transferring the sections into PBS. The stained sections mounted onto gelatinized slides, air dried, dehydrated via ascending alcohol row and coverslipped with Entellan^®^ (Merck, Darmstadt, Germany).

### Immunohistochemistry

Immunohistological approaches were used to assess the number of neurons and the extent of glial scarring around the infusion sites (Hayn and Koch 2015).

Briefly, free-floating brain slices were rinsed three times for 10 min in PBS and transferred in a blocking solution containing 10% normal goat serum (Linaris, Wertheim-Bettingen, Germany) and 0.1% Triton X-100 (Sigma-Aldrich, Steinheim, Germany) for 60 min at RT. Subsequently, sections were incubated at 4 °C for 72 h in blocking solution containing the primary antibody: anti-mouse nuclear neuronal marker (1:1000; NeuN, Millipore, Cat# MAB377 (RRID:AB_2298772)) for neurons, rabbit anti-Iba-1 (1:2000; Wako, Cat# 016–20001 (RRID:AB_839506)) for microglia and rabbit anti-glial fibrillary acidic protein (1:5000, GFAP, Dako, Code no. Z0334, (RRID:AB_10013382)) for astrocytes. Thereafter, sections were rinsed in PBS and incubated in PBS containing 10% bovine serum albumin (PBS-A) at RT for 60 min. Next, sections were incubated for 48 h in PBS-A containing the secondary antibody: biotinylated goat anti-rabbit antibody (GFAP and Iba-1; Dako Cat# E0432, (RRID:AB_2313609)) or biotinylated goat anti-mouse (NeuN; Dako Cat# E0433, RRID:AB_2687905). Afterwards, sections were incubated for 24 h at RT in PBS-A containing Alexa Fluor 594 streptavidin (Sigma-Aldrich, Germany, 1:2000). Sections were mounted on object slides and were counterstained with 0.5% Sudan Black (Acros Organics, Belgium) in 70% ethanol following washing twice in PBS to eliminate autofluorescence. Sections were coverslipped with fluorescence mounting medium (Dako, Glostrup, Denmark).

### Image Analysis

**Fig. 2:**
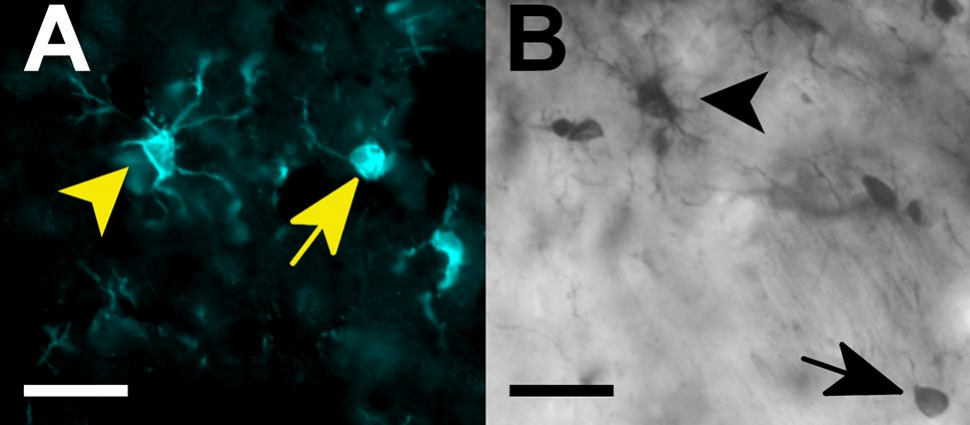
Comparison of cell morphology of cells which were stained immunohistochemically for Iba-1 (microglia, **A**) and for iron via Perls’ Prussian blue with DAB-intensification (**B**). Ramified (black arrowhead) and amoeboid iron-positive cell (black arrow) show the typical morphology of ramified (yellow arrowhead) and amoeboid (yellow arrow) microglia. Scale bar = 25 µm

Fluorescent and light microscopic images of tissue sections from infusion sites were taken using a Zeiss Axioskop II microscope (Zeiss, Göttingen, Germany) and photomicrographs were taken by a digital camera RT slider spot connected to the image analysis software Metamorph 4.6 (Visitron Systems, Puchheim, Germany). For fluorescent images, the appropriate band-pass filter was used for the excitation/emission peaks of Alexa Fluor 594 at 590 nm and 617 nm (red)). Afterwards, the images were transferred to the image processing software FIJI (RRID:SCR_002285; Schindelin et al. 2012). The iron-distribution of Perls-stained sections was examined using the light microscope and qualitatively evaluated by observing the iron-distribution in the tissue in ramified and amoeboid brain microglia (Bishop and Robinson 2001; Fig. 2). To determine the astrocyte density at the infusion sites, the examination method of Hayn and Koch (2015) was used. To obtain binary images, 16-bit images were converted to 8-bit images and contrast was increased by 0.3% followed by the application of an automatic local threshold (method: median; radius: 80 pixels; correction value (*c*): − 30). Astroglia density [%] below the needle tract was calculated for each region of interest (ROI) 450 × 450 μm (0.2 mm^2^).

Because of their heterogenous appearance in shape and diameter, microglia were counted manually with the cell counter plug-in in FIJI. Only ramified and amoeboid cells were considered. Since microglia were expected in the immediate vicinity of the infusion site, Iba-1-positive (Iba-1 +) cells were counted within a ROI of 0.2 mm^2^ underneath the infusion site. To quantify neuronal populations at the infusion sites, 16-bit grey-scale images were converted to 8-bit binary images as mentioned above. “Clotted” neurons were counted using the FIJI “analyse particle” function within a ROI of (0.2 mm^2^) below the infusion site and extrapolated to 1 mm^2^ for analysis. The observer was blind to treatment conditions in all cases.

### Statistical Analysis

All data are expressed as means ± SEM (standard error of the mean). For statistical analysis and graphical presentation of results RStudio version 4.1.2 (RStudio Inc., Boston, MA, USA) was used. Data of T-maze training was analysed via two-way repeated measures (RM) analysis of variance (ANOVA) with factors time (1 week and 4 weeks) and treatment (VEH, FAC and IONP). The parameter correct trials [%] was analysed via three-way RM ANOVA with factors time, treatment, and delay (0 s, 10 s and 30 s). Data of the OF experiments were analysed for the parameters distance travelled [m], activity [%], rearings [n] and time spent in centre [%] via two-way RM ANOVA with factors time and treatment. The habituation to the parameters distance travelled and activity was analysed via two-way RM ANOVA with factors interval (interval 1–6) and treatment. The quantitative data of immunohistochemistry was analysed via one-way RM ANOVA with treatment as factor. Post-hoc Tukey’s *t*-test was performed via emmeans function (emmeans package, (Lenth 2022)) to identify statistically significant differences when significant main effects were detected. For ANOVA the aov function was used (Chambers et al. 1992). The level of significance was set at *p* < 0.05.

## Results

### Nissl-Staining

**Fig. 3:**
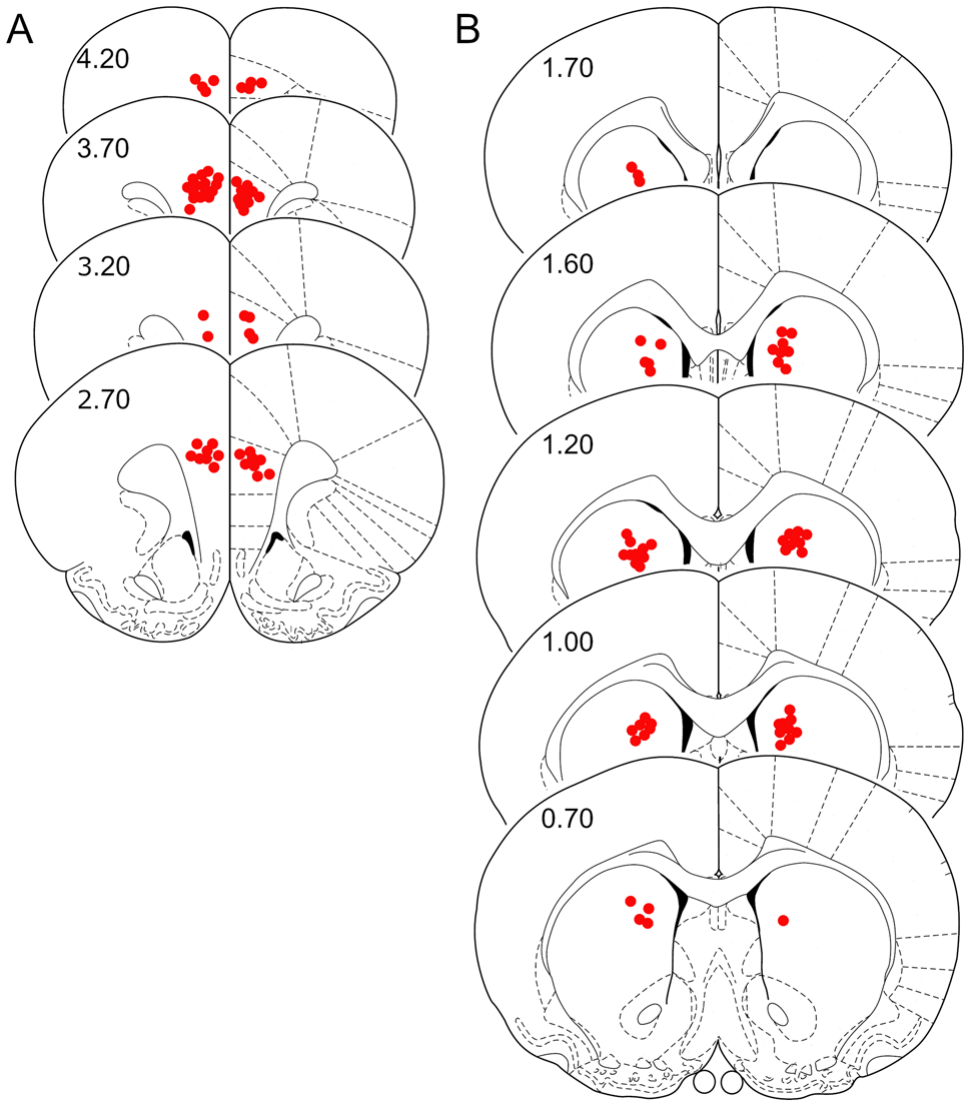
Location of bilateral infusion sites in the medial prefrontal cortex (*n* = 30, **A**) and the caudate putamen (*n* = 27, **B**). Rostral distance (mm) from Bregma is given in numbers. Schematic drawings are taken from the rat brain atlas of Paxinos and Watson (1998)

The analysis of Nissl-stained sections revealed correct cannula placement in the mPFC (*n* = 30; Fig. 3A) and in the CPu (*n* = 27; Fig. 3B). Three rats of the CPu-cohort were excluded from further evaluation because of an extreme statistical outlier due to hyperactive locomotor behaviour (*n* = 1), misplaced cannula (*n* = 1) or structural abnormalities in one brain hemisphere (*n* = 1).

### Distribution of Iron

**Fig. 4:**
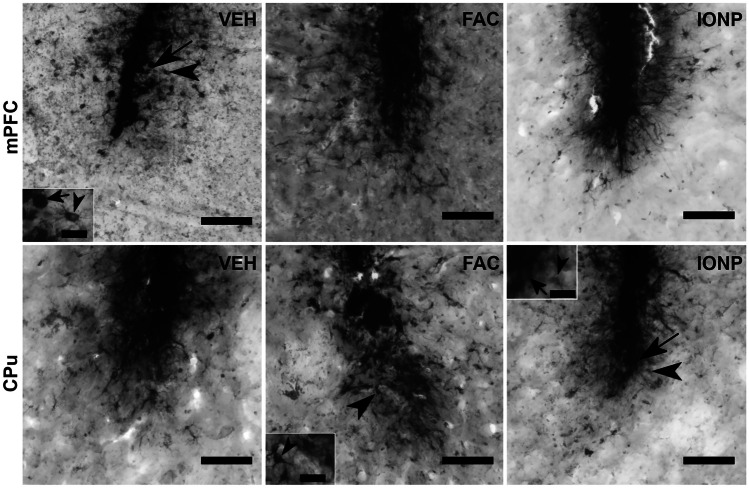
Representative microphotographs of brain sections stained with modified Perls’ Prussian blue method after infusion of vehicle (VEH), ferric ammonium citrate (FAC) or iron oxide nanoparticles (IONP) in the medial prefrontal cortex (mPFC; upper row) and caudate putamen (CPu, lower row). In the mPFC all treatment groups showed iron-positive (iron +) ramified cells (depicted by arrowheads). Amoeboid cells occurred more in the vicinity of the needle tract (depicted by arrows). In the CPu iron + ramified cells in the wider surrounding of the infusion site as well as amoeboid cells were mainly observed in the FAC- and IONP-groups. In the VEH group, iron + cells were mainly close to the needle tract. mPFC, each treatment-group *n* = 10; CPu, each treatment-group *n* = 9. Scale bar = 100 µm; scale bar in the insets = 25 µm

Detection of iron in brain tissue was achieved with Perls’ Prussian Blue and intensification by DAB and was evaluated qualitatively. In both areas, the needle-tracts were clearly visible and iron-positive (iron +) cells were mainly observed in the vicinity of the infusion site, independent of the substance given. Two cell types were observed and distinguished by their morphology: amoeboid (Fig. 4, black arrows) and ramified cells (Fig. 4, black arrowheads).

In all treatment-groups iron + ramified cells were observed in the mPFC. Most labelled cells were observed in FAC- and IONP-treated rats (Fig. 4). Ramified iron + cells were mainly in the vicinity of the needle tract, only in very few cases ramified cells were detected in distant brain parenchyma. Roundish amoeboid iron + cells occurred independent of the treatment and were mainly located at the needle tract.

In the CPu, the strongest presence of iron + ramified and amoeboid cells was observed in animals that received IONP and FAC (Fig. 4). In both groups, iron + ramified cells showed spreading into the brain parenchyma, whereas iron + cells in the VEH-group were more observed close to the needle tract. Amoeboid cells were also primarily observed to a similar extent in the FAC- and in the IONP-group, these cells were mainly in the vicinity of the needle tract.

### Distribution of Astrocytes

**Fig. 5:**
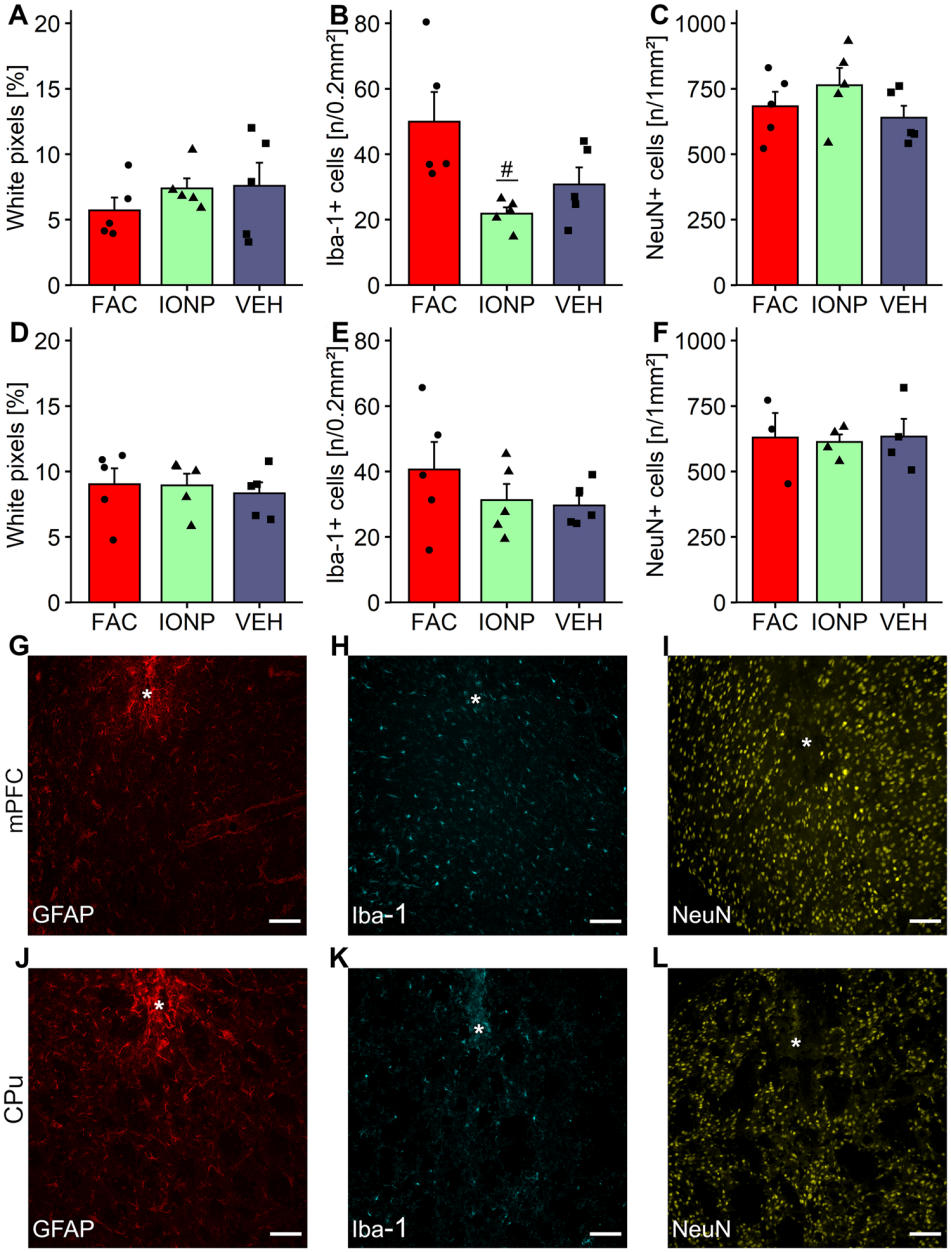
Representative microphotographs and quantitative analysis of immunohistochemical data of rats treated with vehicle (VEH), ferric ammonium citrate (FAC) or iron oxide nanoparticles (IONP) into the medial prefrontal cortex (mPFC) and caudate putamen (CPu). Bar graphs **A** and **D** show the distribution of GFAP-positive (GFAP +) cells at the infusion site in a ROI of 0.2 mm^2^ in the mPFC and CPu, respectively. Bar graphs **B** and **E** show the number of Iba-1-positive (Iba-1 +) cells per 0.2 mm^2^ underneath the infusion site in the mPFC and CPu, respectively. Bar graphs **C** and **F** show the number of neurons per mm^2^ underneath the infusion site in the mPFC and CPu, respectively. Representative images of immunohistochemical staining at the infusion site (marked by the white asterisks) for GFAP, Iba-1 and NeuN are shown in **G**–**I** for the mPFC and in **J**–**L** for the CPu, respectively. Data are means ± SEM, black circles, triangles and squares represent the individual data of rats which receives FAC, IONP and VEH, respectively. The hash denotes a significant difference of the IONP-group compared to the FAC-group. mPFC, each treatment group *n* = 5; CPu, for Iba-1 and GFAP each treatment group *n* = 5, for NeuN VEH and IONP-groups *n* = 4 and FAC-group *n* = 3 (Tukey’s *t*-test; *p* ≤ 0.05); scale bar = 100 µm

Treatment effects on astroglial distribution and reactivity after infusion of VEH, FAC or IONPs were assessed by GFAP-immunohistochemistry. GFAP-expression demonstrates a glial scar formation at the infusion site as expected after such procedures (Irrsack et al. 2021). The one-way ANOVA did not show a significant effect for treatment in the mPFC (*p* = 0.525; *F*_2, 12_ = 0.681; Fig. 5A, G) or CPu (*p* = 0.869; *F*_2, 12_ = 0.142; Fig. 5D, J).

### Distribution of Microglia

To evaluate the presence and reaction of microglia after infusion of VEH, FAC or IONP, we used Iba-1-immunohistochemistry. A one-way ANOVA revealed a treatment-effect in the mPFC (*p* = 0.0207 *F*_2, 12_ = 5.451; Fig. 5B, H), but not for the CPu (*p* = 0.391; *F*_2, 12_ = 1.016; Fig. 5E, K). Post-hoc Tukey’s *t*-test revealed a significant higher number of Iba-1 + cells in the mPFC after FAC-treatment compared to IONP- (*p* = 0,0183) but not to VEH-treatment (*p* = 0.1103).

### Distribution of Neurons

For assessing the number of neurons in the examined areas, brain slices were immunohistochemically stained for NeuN. A one-way ANOVA revealed no significant treatment-effects in the mPFC (*p* = 0.318; *F*_2, 12_ = 1.261; Fig. 5C, I) or CPu (*p* = 0.967; *F*_2, 8_ = 0.033; Fig. 5F, L).

### Open Field

**Fig. 6:**
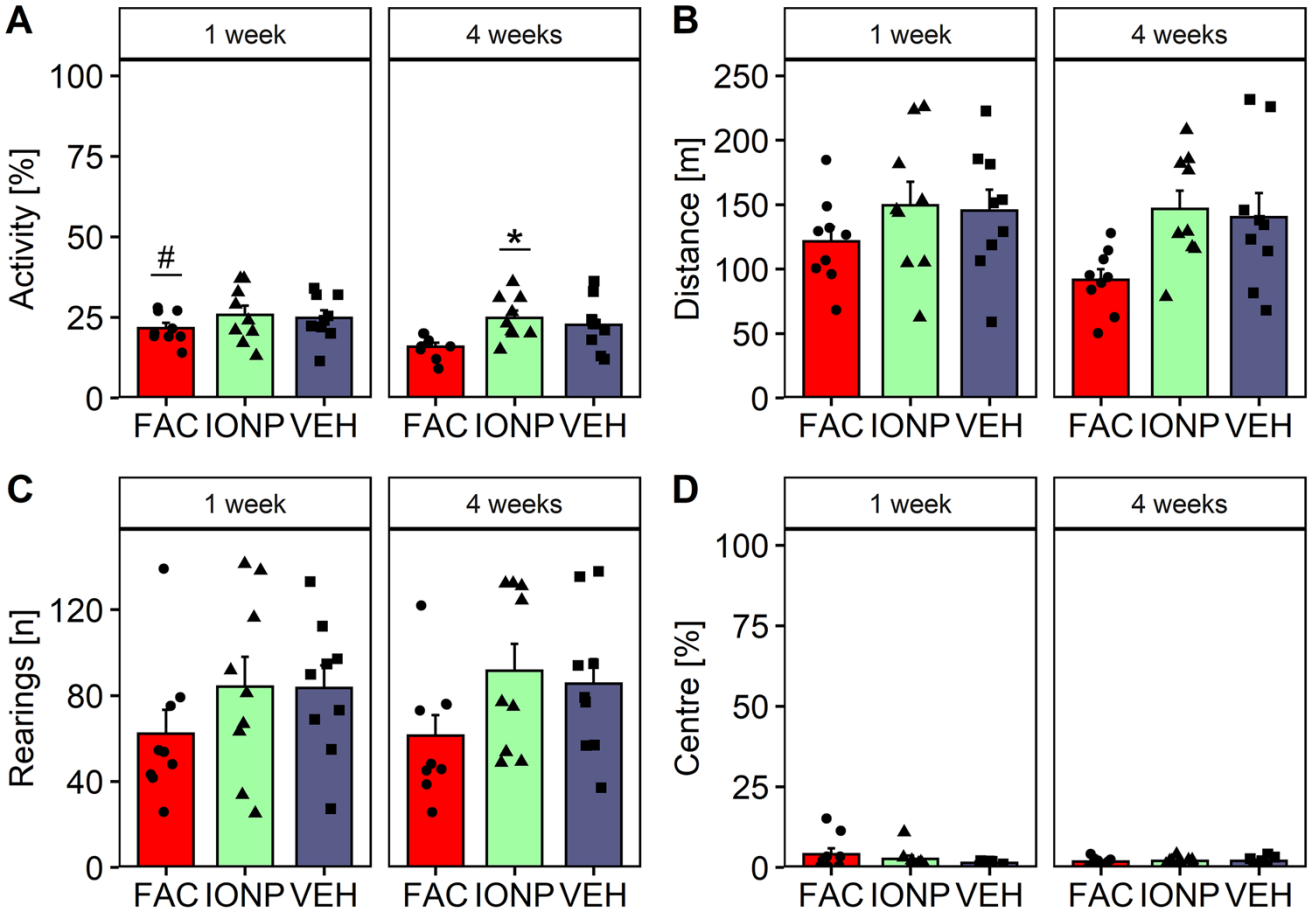
Analysis of parameters of the open field in rats after infusions of ferric ammonium citrate (FAC; *n* = 9), iron oxide nanoparticles (IONP; *n* = 9) or vehicle (VEH; *n* = 9) into the caudate putamen 1-week and 4-week post-infusion. Bar graphs show the means ± SEM of the total motor activity [%] (**A**), of the total distance travelled [m] (**B**), the number of rearings (**C**) and the time [%] spent in centre of the open field box (**D**). Black circles, triangles and squares represent the individual data of rats which receives FAC, IONP and VEH, respectively. The hash denotes a significant difference within the FAC-group between weeks and the asterisk denotes a significant difference between IONP and FAC(Tukey’s *t*-test: *p* ≤ 0.05)

The rats showed no substance-effects in the OF 1 week after treatment. However, infusion of FAC resulted in a decreased total motor activity compared to IONP 4-week post-surgery. Thus, the impact of this effect increased over time within the FAC-group. A two-way RM ANOVA revealed a main effect for factor time (*p* = 0.0179; *F*_1, 24_ = 6.457), but no effect for factor treatment (*p* = 0.0771; *F*_2, 24_ = 2.856) for total motor activity. Post-hoc Tukey’s *t*-tests showed that independent of the treatment the motor activity decreased 4 weeks compared to 1-week post-surgery (*p* = 0.0179). Rats treated with FAC showed a significantly lower activity than rats treated with IONPs (*p* = 0.0211) after 4 weeks. Tukey’s *t*-test revealed that the motor activity in FAC-treated rats decreased significantly after 4 weeks compared to 1 week (*p* = 0.0084) (Fig. 6A). Statistical analysis revealed no significant effects for the factors treatment and time on distance travelled (Fig. 6B). The number of rearings (Fig. 6C) and the time spent in the centre (Fig. 6D) were not affected by any substance at any time. The statistical analysis did not show a significant effect.

**Fig. 7:**
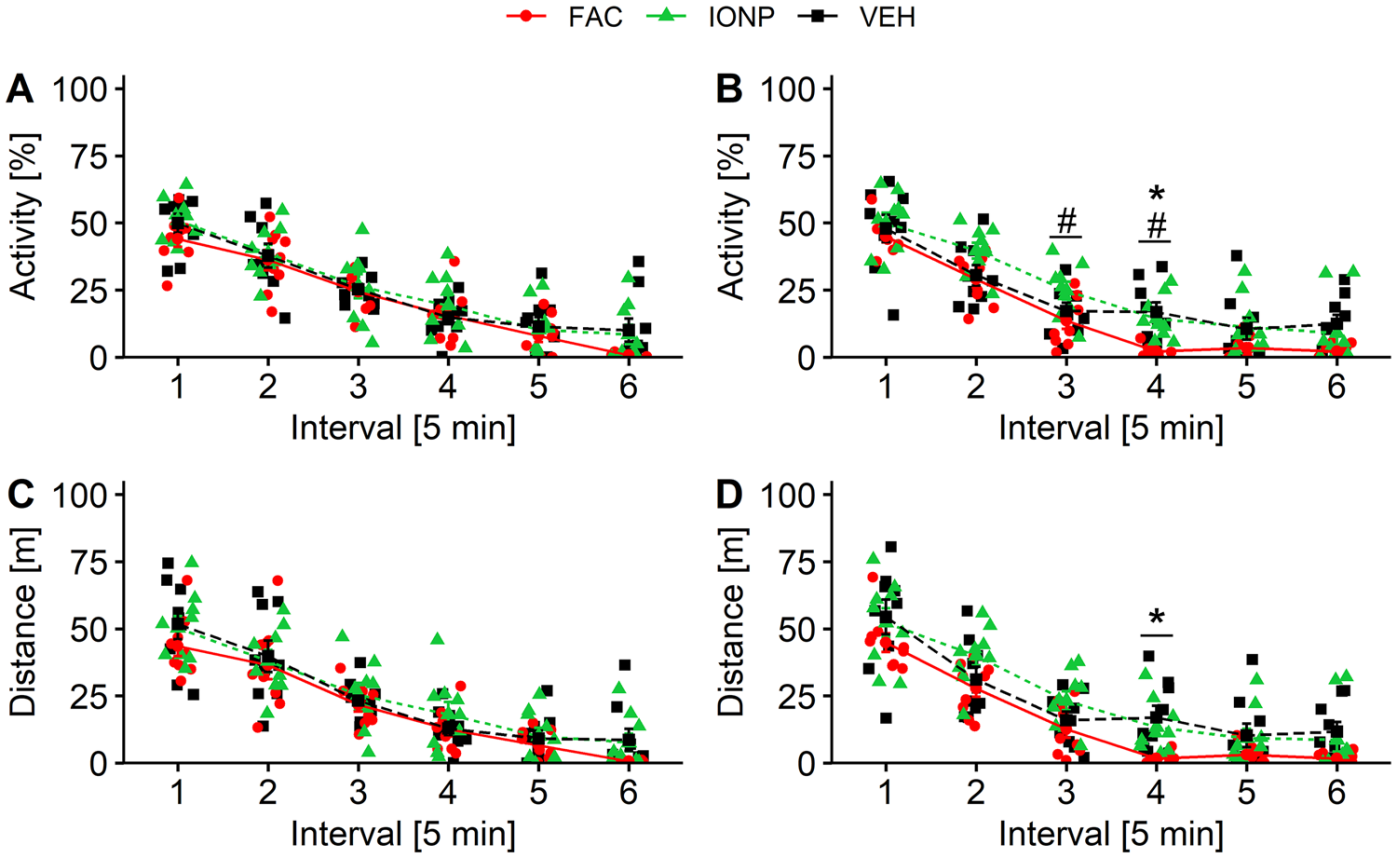
Habituation to the open field in rats after infusions of ferric ammonium citrate (FAC; *n* = 9), iron oxide nanoparticles (IONP; *n* = 9) or vehicle (VEH; *n* = 9) into the caudate putamen as indicated by the decrease in motor activity [%] over a period of 30 min 1-week (**A**) and 4-week (**B**) post-infusion and the decrease in distance travelled [m] over a period of 30 min 1-week (**C**) and 4-week (**D**) post-infusion. The line graph represents the mean ± SEM for 5 min intervals. The red circles, green triangles and black squares represent the individual data of rats that received FAC, IONP and VEH, respectively. Hashes denote a significant difference between the FAC- and IONP-group; asterisks denote significant differences between VEH- and FAC-groups (Tukey’s *t*-test: *p* ≤ 0.05)

As expected, rats habituated to the environment over the 30 min observation time (Gould et al. 2009), the total activity decreased significantly 1-week and 4-week post-surgery. After 1 week, the two-way RM ANOVA revealed a main effect for time-interval (*p* < 0.0001; *F*_5, 120_ = 127.357), but not for treatment (*p* = 0.421; *F*_2, 24_ = 0.896; Fig. 7A). Similar results were found for the total distance travelled. The statistical analysis revealed a significant effect for factor time-interval (*p* < 0.0001; *F*_5, 120_ = 94.885), but not for treatment (*p* = 0.399; *F*_2, 24_ = 0.955; Fig. 7C). For the total activity in the OF 4-week post-surgery the statistical analysis showed the strongest effect of time-interval (*p* < 0.0001; *F*_5, 120_ = 108.243) and treatment (*p* = 0.0181; *F*_2, 24_ = 4.761; Fig. 7B). FAC-treated rats showed a significantly lower average activity than IONP-treated rats (*p* = 0.0178) 4-week post-surgery. Four-week post-surgery FAC-infused rats showed significant lower activity during interval 3 (*p* = 0.0436) and 4 (*p* = 0.0245) compared to IONP and during interval 4 compared to VEH (*p* = 0.0053). For the parameter total distance travelled the statistical analysis revealed main effects for time-interval (*p* < 0.0001; *F*_5, 120_ = 88.028) and treatment (*p* = 0.0241; *F*_2, 24_ = 4.369; Fig. 7D) 4-week post-surgery. FAC-treated rats covered a significant shorter average distance than IONP-treated rats (*p* = 0.0322) 4-week post-surgery. During interval 4 FAC-treated rats travelled significant less than VEH-treated rats (*p* = 0.0144).

**Fig. 8:**
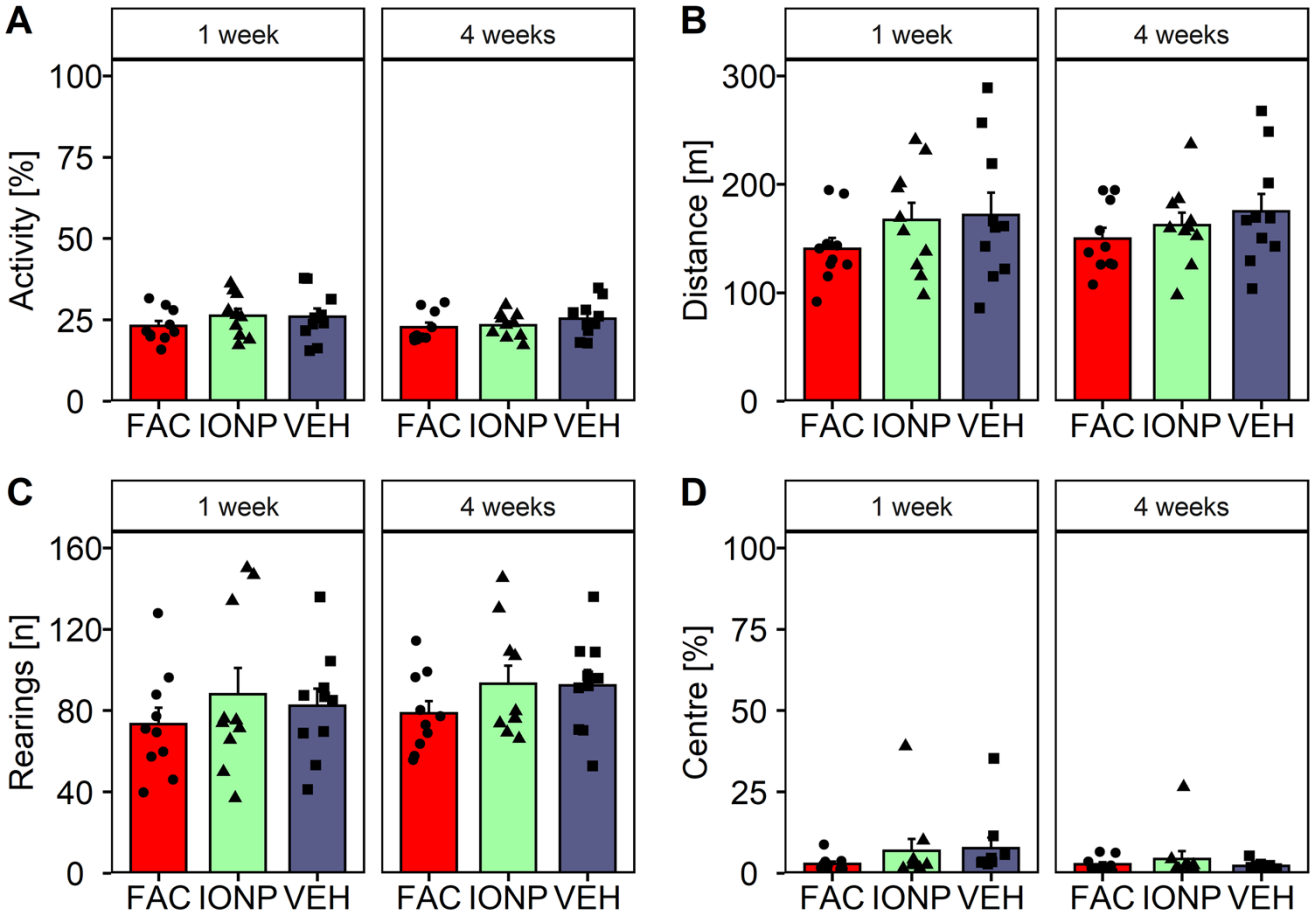
Locomotor activity in rats after infusions of ferric ammonium citrate (FAC; *n* = 10), iron oxide nanoparticles (IONP; *n* = 10) or vehicle (VEH; *n* = 10) into the medial prefrontal cortex 1-week and 4-week post-treatment. Bar graphs show the means ± SEM of the total motor activity [%] (**A**), of the totals distance travelled [m] (**B**), of the number of rearings (**C**) and the time [%] spent in centre of the open field (**D**). Black circles, triangles and squares represent the individual data of rats which receives FAC, IONP and VEH, respectively (Tukey’s *t*-test: *p* ≤ 0.05)

The mPFC-cohort was tested in the OF as well. The treatments had no impact on total motor activity (Fig. 8A), distance travelled (Fig. 8B), total number of rearings (Fig. 8C) or time spent in the centre (Fig. 8D). The statistical evaluation revealed no significant effects, suggesting no effect on locomotor behaviour.

### T-Maze Alternation Task

**Fig. 9:**
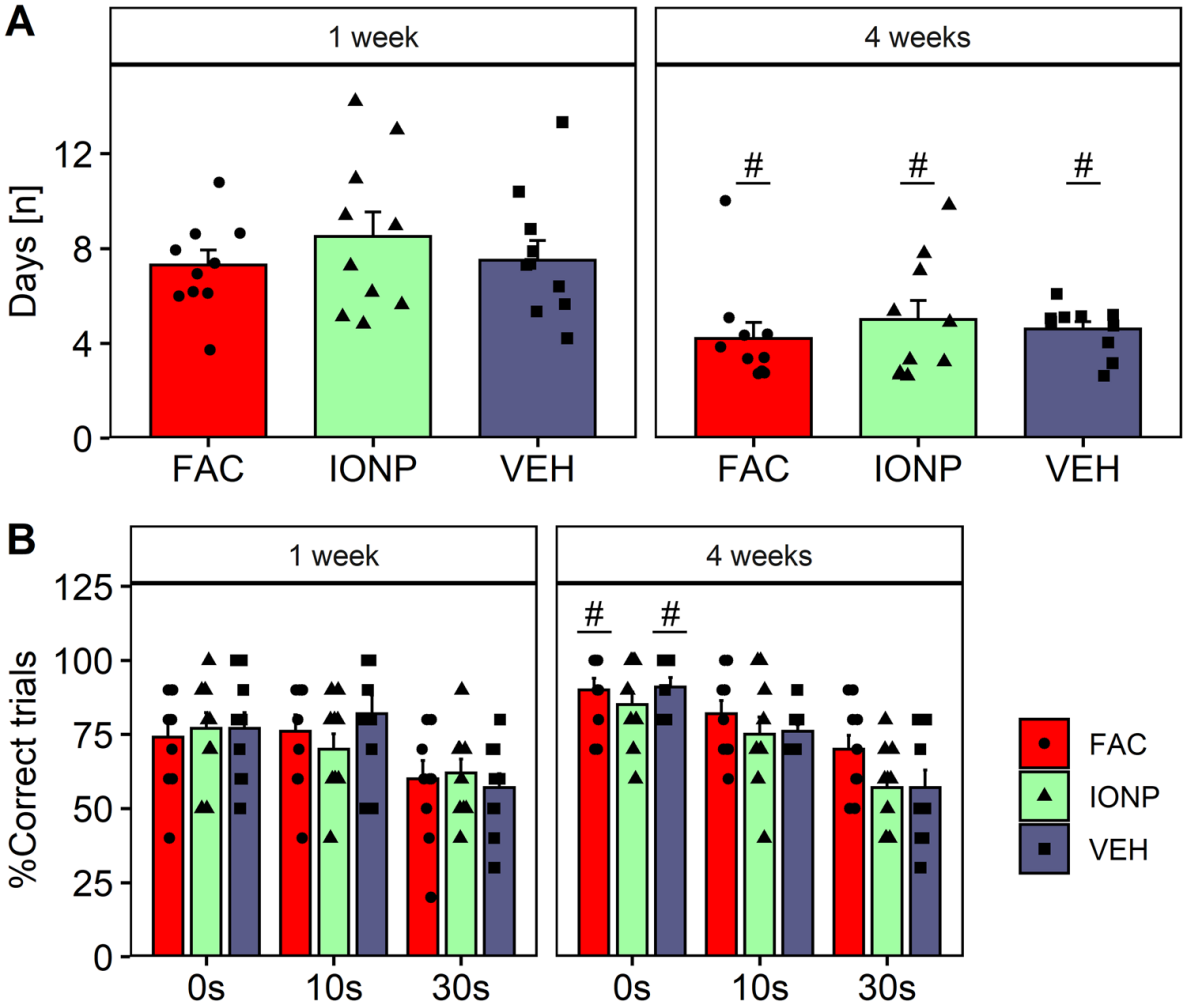
Effects of ferric ammonium citrate (FAC; *n* = 10), iron oxide nanoparticles (IONP; *n* = 10) or vehicle (VEH; *n* = 10) in the medial prefrontal cortex 1-week and 4-week post-infusion on working memory performance in the delayed alternation T-maze task. **A** The number of training-days needed to reach the criterion of 80% correct responses for three consecutive or on four of five consecutive days with start of training 1-week and 4-week post-infusion. **B** The correct trials [%] during the time-block 1-week and 4-week post-surgery. Bar graphs show mean ± SEM. Black circles, triangles and squares represent the individual data of rats which receives FAC, IONP and VEH, respectively. Hashes denote differences compared to 1 week within a treatment group (Tukey’s *t*-test: *p* ≤ 0.05)

Infusion of IONPs into the mPFC had no effect on the training performance or relearning of the task after 4 weeks. In the test session, only the VEH and FAC groups showed improved performance after 4 weeks during the 0 s delay, but there were no substance effects. A two-way RM ANOVA revealed no treatment effects for the training sessions (*p* = 0.242; *F*_2, 27_ = 1.498), but a significant effect for the factor time (*p* = 0.000146; *F*_1, 27_ = 19.511; Fig. 9A). Post-hoc Tukey’s *t*-test showed that the time-effect was independent from treatment (*p* = 0.0001). Rats treated with VEH (*p* = 0.0272), FAC (*p* = 0.0189) or IONPs (*p* = 0.0089) needed significantly less days to reach the criterion 4-week post-surgery compared to the training which started 1-week post-surgery indicating that relearning of the strategy was not disturbed by treatment.

For the test-performance during the 1-week and 4-week blocks, a three-way repeated measures ANOVA showed a main effect for the factor delay (*p* < 0.0001; *F*_2, 54_ = 50.77), a significant interaction between delay and time (*p* = 0.0194; *F*_2, 54_ = 4.245) suggesting that the performance changed with repeated test-session after 4 weeks, but no main effects for the factors treatment and time were detected. Multiple comparisons by Tukey’s *t*-test revealed that rats treated with VEH (*p* = 0.0328) or FAC (*p* = 0.0152) showed a significant improvement of performance after 4 weeks compared to 1w at the 0 s delay (Fig. 9B).

## Discussion

We previously showed that intracerebral administration of IONPs to the mPFC, CPu and dorsal hippocampus had no neurotoxic effects (Irrsack et al. 2021). Nevertheless, IONPs may impair brain function without overt structural lesions, for example by affecting physiological processes, which may have an impact on behaviour (Manickam et al. 2019). Therefore, the present study investigated the effects of local microinfusions of IONPs into the rat’s mPFC and CPu on cognitive and motor behaviour. While intracerebral administration of IONPs had no adverse consequences, the infusion of FAC into the CPu resulted in a decrease in total motor activity in the OF 4-week post-infusion compared to 1w post-infusion and the injection into the mPFC caused a local immune response. However, working memory performance in a delayed alternation task in the T-maze was not affected by FAC-administration into the mPFC at any time-point.

The mPFC is implicated in working memory in rodents and is also involved in the modulation of motor behaviour (Lacroix et al. 1998; Groenewegen 2003; Kehr et al. 2018). Lesion or deactivation of the mPFC leads to an impaired performance in the delayed alternation tasks, e.g. in the T-, the figure-eight-maze and in an operant-chamber (Sánchez-Santed et al. 1997; Yoon et al. 2008; Horst and Laubach 2009; Tsutsui et al. 2016). Neither the motor behaviour and emotional state of rats in the OF nor the working-memory performance in the T-maze was affected by any of the substances. In the present study, the only significant impact was caused by the infusion of FAC into the mPFC, which led to an increased number of Iba-1 + cells compared to IONP-treated rats. Both FAC and IONPs were taken up by microglia cells indicated by increased iron + ramified cells. Microglia may play a neuroprotective role by taking up iron (Bishop et al. 2011; Urrutia et al. 2013; Olmedo-Díaz et al. 2017), migrate to the region of impact and phagocytosis of apoptotic cells and cell debris to maintain neuronal functioning (Galloway et al. 2019). We have previously reported an increased neurodegeneration 1 week after FAC-infusion into the mPFC as indicated by an increased number of Fluorojade-C positive cells, and that the infusion itself caused neuronal loss in the mPFC after 4 weeks (Irrsack et al. 2021). Thus, we conclude that the strong microglial immune reaction after FAC-infusion protected cortical neurons from cell death and facilitated a proper working memory performance in the T-maze.

As primary input region of the basal ganglia the dorsal striatum plays a pivotal role in motor control and executive functions (Florio et al. 2018) and is known to be involved in the habituation to novel environments (Struthers et al. 2005). Therefore, we assessed total motor activity, distance travelled, number of rearings and time spent in the centre of rats in the OF. After infusion of IONPs no effects were found in the OF at any time-point and any of the investigated parameters, suggesting that no major striatal damage had occurred after our treatments.

Rats treated with FAC showed a rapid habituation to the OF 4-week post-infusion indicated by lower motor activity and distance travelled at fourth interval, resulting in a decrease of the total motor activity compared to 1-week post-surgery. Previously, Huang et al. (2019) found similar results after chronic oral administration of ferric citrate in mice in a dose-dependent way. They hypothesized that this was caused by neurotoxic effects of iron on dopaminergic neurons. The striatum is physiologically rich in iron (Connor and Menzies 1995; Cheli et al. 2020; Wang et al. 2022) and an imbalance of striatal iron concentrations can lead to alterations in dopamine metabolism (Kim and Wessling-Resnick 2014; Imam et al. 2015). However, the extent to which intracerebral administration of FAC affects dopamine metabolism was not examined in the present study and needs to be clarified by further studies.

The results of studies on the influence of IONPs on the behaviour of rodents have been heterogeneous. Previous studies revealed disturbed motor coordination, locomotion and memory deficits in the Morris water-maze after chronic oral administration of IONPs (Manickam et al. 2019) and i.p. injections of IONPs (Dhakshinamoorthy et al. 2017). The authors found decreased levels of antioxidants, suggesting neuronal oxidative stress, increased acetylcholinesterase activity, lactate dehydrogenase leakage and demyelination in the frontal cortex, hippocampus and cerebellum, leading to neurobehavioural impairments (Dhakshinamoorthy et al. 2017). Moreover, Manickam et al. (2019) found altered monoamine levels and mitochondrial dysfunctions leading to energy depletion and neuronal dysfunction causing cognitive impairments. Another study revealed that intranasal instillation of IONPs in rats reduced exploratory behaviour and locomotion in the hole-board test (Minigalieva et al. 2023). However, Askri et al. did not find effects on exploratory behaviour, spatial reference memory, locomotion or emotions after acute oral treatment (Askri et al. 2019), and no effects on spatial reference memory or on emotional behaviour after chronic intranasal application of IONPs, despite an increase of dopamine and norepinephrine levels (Askri et al. 2018). The contrasting results reported in the different studies regarding behavioural consequences of an IONP exposure as well as molecular and cellular parameters are likely to be caused by differences in study design and the type of substance administration, but also using different types of IONPs. For instance, the coating material can have an influence by modifying physicochemical parameters such as the size, zeta-potential, shape and curvature of IONPs and, therefore, will strongly influence the IONP-biomolecule-interaction (Nel et al. 2009; Yarjanli et al. 2017; Bardestani et al. 2021; Salehipour et al. 2021). While the iron core is responsible for the magnetic properties, the ligand coat stabilises the IONPs (Laurent et al. 2008). Previous studies demonstrated for example differences between the effects of non-coated and dextran-coated IONPs in zebrafish larvae (de Oliveira et al. 2017) or differences between different coating materials like dextran and gold after intrastriatal infusions in rats concerning the diffusion into the tissue (Wang et al. 2011a).

To our knowledge, this is the first study to use local intracerebral infusions of IONPs into selected brain areas to investigate possible effects on working memory and motor behaviour. In the present study, local administration of IONPs into the mPFC and CPu showed no behavioural effects as well as no histological effects on neural viability. Furthermore, it has already been shown in cell culture that neurons can take up IONPs without affecting neuronal viability (Petters and Dringen 2015). As microglia can efficiently internalize IONPs (Petters et al. 2016), we assume that most of the IONPs injected into brain were taken up by microglia before they could harm neurons, which is supported by the increased abundance of ramified iron + cells.

To conclude, IONPs injections into the brain did not cause adverse effects, neither at the cellular nor at the functional level. Similar to the VEH-group, IONP-treated rats showed stable working memory performance in the T-maze and, in contrast to FAC, did not show no decrease in motor activity in the OF. Neurons were not damaged and most of the IONPs were probably taken up by microglial cells. These findings support our in vivo findings for the mPFC and CPu that IONPs have low toxic potential (Irrsack et al. 2021). Nevertheless, further behavioural studies should be performed in this regard to be able to exclude potential dysfunction of the investigated areas over time. Therefore, in addition to neuronal viability and the reaction of glial cells, a possible influence of the treatment with IONPs on the levels of certain biomolecules such as the monoaminergic system should be investigated in this context in order to be able to exclude any adverse effects to brain functioning (Manickam et al. 2019).

## Data Availability

Data will be made available on request.
